# Therapeutic Effects of Cold Atmospheric Plasma on Solid Tumor

**DOI:** 10.3389/fmed.2022.884887

**Published:** 2022-05-13

**Authors:** Tianhao Min, Xin Xie, Kaijie Ren, Tuanhe Sun, Haonan Wang, Chengxue Dang, Hao Zhang

**Affiliations:** ^1^Department of Surgical Oncology, The First Affiliated Hospital of Xi'an Jiaotong University, Xi'an, China; ^2^Department of Nuclear Medicine, The First Affiliated Hospital of Xi'an Jiaotong University, Xi'an, China

**Keywords:** cold atmospheric plasma, solid tumor, reactive oxygen and nitrogen species, CAP, ROS, RNS

## Abstract

Cancer is a devastating disease, and there is no particularly effective treatment at present. Recently, a new treatment, cold atmospheric plasma (CAP), has been proposed. At present, CAP is confirmed to have selective killing effect on tumor by many studies *in vitro* and *in vivo*. A targeted literature search was carried out on the study of cold atmospheric plasma. Through analysis and screening, a narrative review approach was selected to describe therapeutic effects of cold atmospheric plasma on solid tumor. According to the recent studies on plasma, some hypothetical therapeutic schemes of CAP are proposed in this paper. The killing mechanism of CAP on solid tumor is expounded in terms of the selectivity of CAP to tumor, the effects of CAP on cells, tumor microenvironment (TME) and immune system. CAP has many effects on solid tumors, and these effects are dose-dependent. The effects of optimal doses of CAP on solid tumors include killing tumor cells, inhibiting non-malignant cells and ECM in TME, affecting the communication between tumor cells, and inducing immunogenic death of tumor cells. In addition, several promising research directions of CAP are proposed in this review, which provide guidance for future research.

## Background

According to the latest global cancer burden data presented by the World Health Organization (WHO) and International Agency for Research on Cancer (IARC), in 2020, the number of new cancer cases worldwide rose to 19.29 million, affecting 10.06 million males and 9.23 million females, and the number of worldwide cancer deaths rose to 9.96 million, affecting 5.53 million males and 4.43 million females. One in five people in the world develops cancer in their lifetime, and one in eight men and one in 11 women die of cancer (1). These data convincingly show that cancer has become a major disease that cannot be ignored. Recently, great progress has been made in cancer treatment, and many new methods have been used to treat cancer at any stage (2–4). However, new research is needed to improve cancer treatment efficacy. The latest developments in oncology include combined therapy, which generally affects several tumor cell mechanisms either simultaneously or consecutively to achieve a better therapeutic effect. Although many studies have focused on targeted therapy and immunotherapy (5–7), other studies have focused on promising cold atmospheric plasma (CAP)-related therapy. CAP has been proven to exert a significant lethal effect on tumor cells (8–10).

CAP, also known as cold physical plasma or nonthermal plasma, is a gas that is partially ionized upon discharge and simultaneously triggers the production of many reactive oxygen species (ROS) and reactive nitrogen species (RNS) (together known as RONS). (11) CAP is composed of transient, high-energy and chemically active substances (electrons, ions, metastable state and free radicals). It exhibits radiation, gas dynamics and electric field characteristics (12). It usually is applied at body temperature to produce many ROS in the gas phase, including hydroxyl radicals (OH), superoxide radicals (O2-), ozone (O3), atomic oxygen and singlet δ oxygen, and RNS, including peroxynitrite (ONOO), nitrogen dioxide radical (NO2) and nitric oxide (NO) (13, 14). RONS and the oxidative stress responses that they trigger have been confirmed to be related to the killing of tumor cells (8, 9, 15, 16); the specific mechanism is described herein.

The solid tumor formation is a complex processes involving a variety of cells (such as endothelial cells, fibroblasts, inflammatory cells and immune cells) and acellular components (such as extracellular matrix (ECM) factors and secretory factors); collectively, these cellular and extracellular factors are referred to as the tumor microenvironment (TME) (14). To study the effect of CAP on solid tumors, we should not limit the investigation into its effects on tumor cells but should also consider the effects of the TME. An increasing number of people have realized the importance of the TME in the treatment of solid tumors, especially in regulating tumor growth and deterring chemotherapeutic drug resistance (17).

This review discusses the role of CAP in solid tumors. It elaborates the following aspects of CAP as a tumor treatment: feasible CAP treatment methods reported in recent studies; effective CAP components, selective CAP effects on tumor cells; the mechanisms by which CAP induces oxidative damage in tumor cells, affects the TME, and causes immunogenic cell death (ICD); and the research prospects for CAP in tumor therapy.

## Cap Devices And Hypothetical Cap Treatments In Tumors

### CAP Devices

Many kinds of gases, such as argon, helium, nitrogen, oxygen, air, and mixed gases, are currently used in CAP (18). Different methods and equipment for producing CAP have been developed on the basis of different biomedical conditions (19). Two main types of methods are used directly and indirectly produce plasma, namely, dielectric barrier discharge (DBD) and plasma jets (20). Many laboratories have developed their own specific CAP production equipment because it is easy and inexpensive to make. However, the use of different equipment makes comparing the results of plasma therapy under the same conditions difficult. Therefore, the standardization of CAP devices is particularly important. Currently, the following types of medical products have been Conformite Europeenne (CE) certified: the DBD device PlasmaDerm^®^ VU-2010 (CINOGY Technologies GmbH), the atmospheric pressure plasma jet (APPJ) kINPen^®^ MED (INP Greifswald/neoplas tools GmbH), and the SteriPlas^®^ (Adtec Ltd., London, United Kingdom). (19)These devices have laid the foundation for the application of plasma therapy to solid tumors.

To standardize plasma sources, Mann et al. released DINSPEC91315 in 2014, which specified physical and technical standards (temperature, thermal power, radiation emission, ultraviolet radiation intensity, gas emission, and current flow) of plasma sources for biomedical applications, as well as basic criteria for characterizing biological plasma effects (namely, the effects on microorganisms *in vitro*) (21). DINSPEC91315 laid the foundation for biomedical research on plasma equipment. However, the standard is developed through existing standards and guidelines (DINENISO12100, DINEN60601-1, DINEN60601-1-6 and DINEN60601-2-57), and therefore, it does not define specific limits, as the evaluation of different parameters depends to a large extent on the expected effectiveness and application of the plasma equipment. Other characteristics of medical plasma equipment need to be added to the standards, including physical evaluation criteria, biological evaluation standards and chemical substance detection. The physical standards include gas temperature, gas composition, heat output, electromagnetic radiation and leakage current, while biological standards include the effects on microorganisms, eukaryotic cells and cancer cells. Furthermore, the composition of ROS and RNS and the effect of pH need to be evaluated through chemical detection (22). Collectively, specification of these parameters may improve the DINSPEC91315 and lead to a unified international standard for medical plasma equipment.

### Hypothetical CAP Treatment of Tumors

Recently, CAP has been applied to a variety of the clinical situations, especially in the field of dermatology, where it has been used for disinfection, the treatment of atopic eczema, ichthyosis/epidermal barrier defects, wound healing promotion, and scar and herpes zoster treatments (19, 23). CAP therapy of solid tumors is not yet being applied in the clinic [only a few studies have reported CAP cancer applications, and they describe CAP applied to the palliative treatment of end-stage head and neck cancer and grossly contaminated tumor ulcerations (24, 25)]; it is only in the laboratory research stage (26). In recent research, CAP treatment is mainly categorized into two types: direct treatment, that is, the use of a CAP-generating device to directly affect tumors, and indirect treatment, that is, the use of a CAP-generating device to treat a solvent that is subsequently applied to tumors.

**Figure 1:**
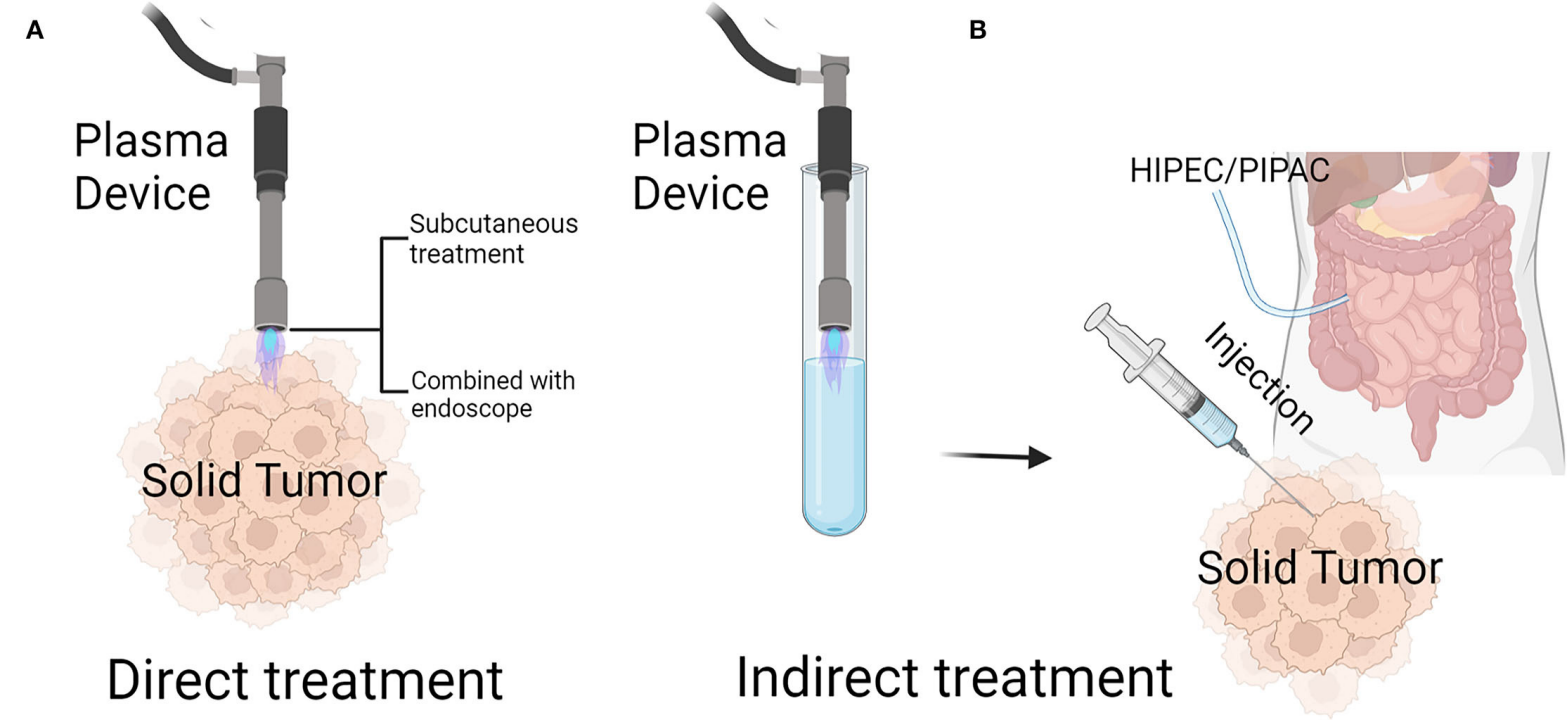
Treatment of solid tumors by cold atmospheric plasma (CAP). **(A)** Direct treatment of CAP can be done by spraying subcutaneous tumors through the skin, or in combination with endoscopes, by irradiating solid tumors through the digestive tract or abdominal cavity. **(B)** Indirect treatment of CAP: after activating a specific liquid with a CAP device, the plasma-activated liquid can be injected directly into solid tumors, or intraperitoneal perfusion can be carried out by HIPEC/PIPAC. HIPEC, Hyperthermic Intraperitoneal Chemotherapy; PIPAC, Pressurized Intraperitoneal Aerosol Chemotherapy. Created by Biorender.

These two treatment modalities have unique advantages and disadvantages. Because many ROS are short-lived and therefore only have a very short free diffusion path length, it is hard for them to reach their biological target (27). However, the applications for this treatment are relatively few, and recent research on direct CAP use has mainly been focused on subcutaneous tumor treatment (Figure 1A). We are committed to making plasma devices more portable for the treatment of other tumors. Recently, a team has developed a small and flexible plasma gun, and animal experiments have been carried out with it to prove CAP treatment efficacy against melanoma (28). We assume that a flexible microplasma gun combined with endoscopy (29) will be a promising device for treating tumors such as gastric, colorectal, lung or abdominal cavity metastases. As a consequence of indirect CAP treatment, the long-lived species hydrogen peroxide and nitrite, present in CAP-activated liquids, can interact in a complex set of reactions and generate the short-lived species singlet oxygen. Singlet oxygen then can inactivate catalase that is specifically present on the surface of tumor cells, but not on nonmalignant cells. As a consequence, the tumor cells start to generate secondary singlet oxygen through the interaction between hydrogen peroxide and peroxynitrite that are generated by the tumor cells themselves. This autoamplificatory mechanism leads to the inactivation of the protective membrane-associated catalase of tumor cells and thus allows specific ROS/RNS-mediated apoptosis-inducing signaling specifically in tumor cells (30–32). Indirect plasma therapy can be used in more types of tumors than direct plasma therapy because it can be injected into solid tumors or added to an abdominal lavage to treat disseminated peritoneal carcinomatosis (11) (Figure 1B).

With respect to the safety of plasma therapy, direct therapy has been safely used in dermatology applications, as mentioned above. In addition, although no clinical safety tests have been performed on indirect plasma therapy, many teams have verified the safety of plasma-activated water (PAW) in animal experiments. For example, a toxicity assessment with CD1 mice confirmed that long-term exposure to PAW had no negative effect on homeostasis, did not induce functional or histological changes in important organs, did not cause changes in hematological or biochemical blood parameters, and did not promote inflammation. (33) Xu et al. also proved indirect therapy safety by treating immunodeficient nude BALB/mice with an oral lavage of PAW daily for 2 weeks (34). In addition, Xu et al. proved that PAW can be safely injected into the bone marrow of New Zealand rabbits without inducing obvious toxicity (35).

## Active Cap Components And The Mechanisms By Which They Kill Tumor Cells

According to recent research on the direct or indirect treatment of CAP, the active components produced by CAP are mainly RONS, which can cause oxidative damage in cells and lead to cell death (36–38). Other active CAP components include freely charged particles, ultraviolet radiation and electric fields, but the mechanisms by which these active components affect tumors have not been extensively studied. The molecular mechanism by which CAP induces oxidative damage to tumor cells is described in detail below.

### Oxidative Damage Mechanism of Plasma-Derived RONS and Its Selectivity Toward Tumor Cells

In general, ROS refers are composed of oxygen and have active properties in an artificial or natural environment. Herein, we discuss ROS produced by plasma. Many valuable studies have proven that ROS produced by plasma include hydrogen peroxide (H_2_O_2_), hydroxyl radicals (OH), superoxide radicals (O${}_{2}^{-}$), O_3_, atomic oxygen and singlet δ oxygen (13, 39). RONS, especially short-lived RONS, have been proven to be the main active agents exerting biochemical and molecular biological effects through direct and indirect plasma therapy (10, 40, 41). ROS can cause oxidative modification in all biomolecules. Cells utilize several antioxidant mechanisms to prevent ROS/RNS-mediated damage. For non-malignant cells, ROS are generally derived from mitochondrial respiration and external damage (radiation, ionization, etc.). To prevent ROS-induced oxidative damage to cells, cells activate an antioxidant defense system, which is categorized into enzymatic (catalase, glutathione peroxidase and superoxide dismutase) and nonenzymatic (vitamin E, vitamin C, glutathione, β-carotene, urate, bilirubin flavonoid, etc.) forms (42). During oncogenesis, most malignant cells express NADPH oxidase (NOX) in their membrane and thus generate extracellular superoxide anions. These will dismutate and form hydrogen peroxide, which is used as a proliferation signal by the malignant cells. Extracellular superoxide anions as well as hydrogen peroxide of malignant cells also establish specific ROS/RNS-mediated apoptosis-inducing signaling, based on the HOCl and the NO/peroxynitrite signaling pathway. Therefore, efficient tumor progression requires the expression of membrane-associated catalase and superoxide dismutase (SOD), which interfere with ROS/RNS-mediated apoptosis-inducing signaling (31, 32, 43). Trachootham et al. suggested that apoptosis is induced when the intracellular oxidative pressure is exceeding the antioxidant potential of tumor cells. As the experimental findings by Trachootham et al. on which this concept is based had been established through the study of transformed cells, i.e., early stages of carcinogenesis that are not protected by membrane-associated catalase, their study neglected the protection of tumor cells toward exogenous ROS and RNS through membrane-associated catalase (44). Their concept is contrasted by the findings established with bona fide tumor cells, as discussed by Bauer et al. (30, 31, 43). Nitrite, a long-lived RNS, and hydrogen peroxide, a long-lived ROS interact in a synergistic interaction, based on the generation of peroxynitrite and resulting in the formation of singlet oxygen (30, 31, 43). Singlet oxygen produced in this way can inhibit tumor cell membrane-associated catalase and SOD, resulting in the flow of CAP-derived ROS/RNS into tumor cells. (31) RNS-derived nitric oxide (NO) regulates posttranslational modifications, S-nitration and genome-wide epigenetic modifications, which can either promote or inhibit tumorigenesis (32). The fate of cancer cells is RNS concentration-dependent. In tumor cells with high levels of NO, the extra RNS produced by CAP may overwhelm the system and shift the role of NO from promoting tumor formation to inhibiting tumor formation (38).

To determine the selectivity of CAP effects on tumor cells, a team compared tumor cells with homologous normal cells. The results presented in their review showed that, among 33 evaluated cell lines, 26 cell lines showed strong CAP selectivity, five showed weak CAP selectivity, and only two showed negative CAP selectivity (45). This study proved the selectivity of CAP therapy for most tumors. Therefore, herein, we review previous studies, compare the differences in plasma-derived RONS uptake and clearance between tumor cells and nonmalignant cells and explain the mechanism of the selective CAP effect on tumor cells.

With regard to the intake of RONS, many studies have clarified the selective mechanism of the CAP effect on tumor cells, including the cholesterol content and the aquaporin (AQP) expression in the tumor cell membrane.

**Figure 2:**
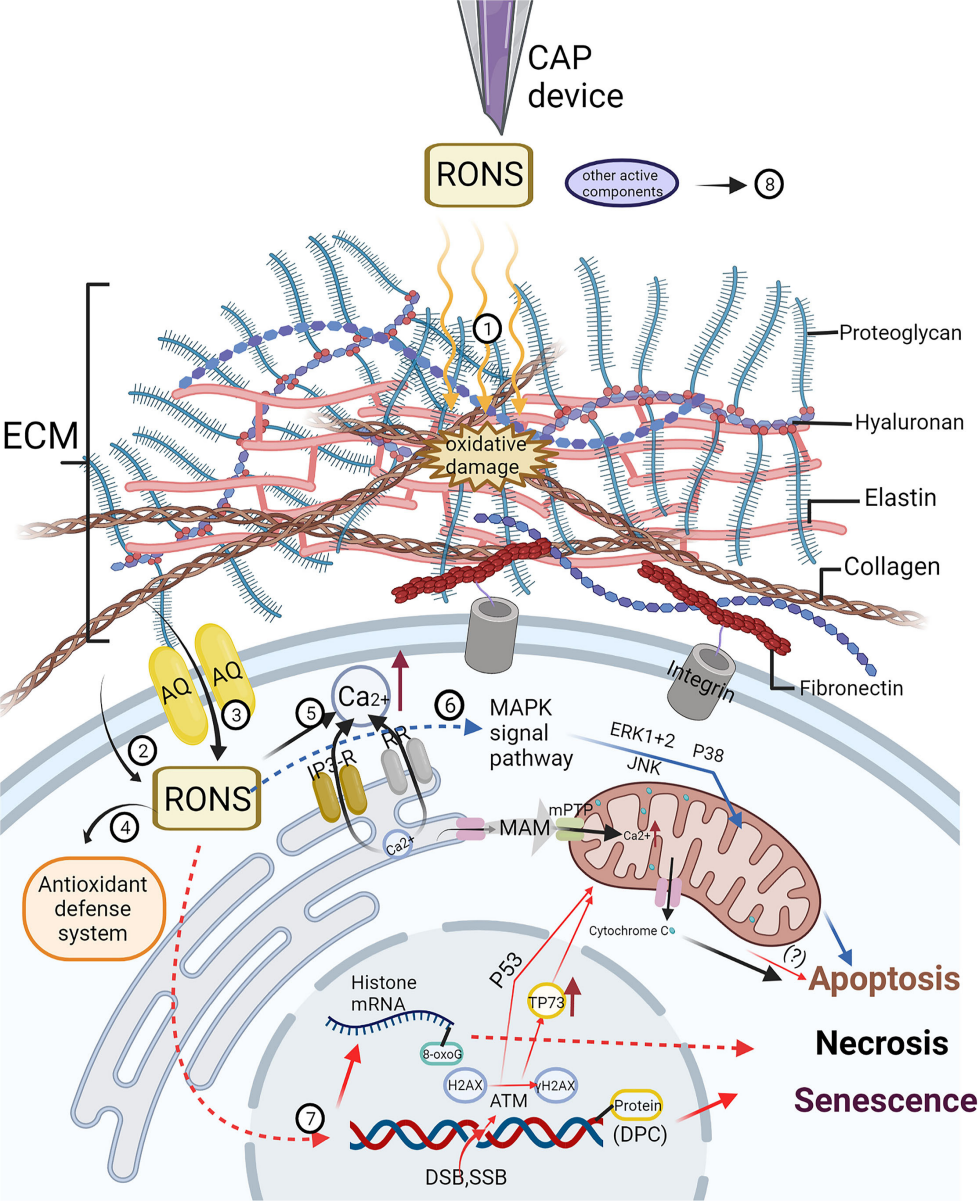
The effect of cold atmospheric plasma (CAP) on extracellular matrix (ECM) and its molecular mechanism on tumor cells have been studied at present. (1) CAP can inhibit tumor progression by oxidizing or destroying the structure of extracellular matrix (ECM), which includes collagen, hyaluronan, fibronectin, integrin and so on. (2) The cholesterol content of tumor cell membrane is lower than that of non-malignant cells, and it is easier for CAP-derived reactive oxygen and nitrogen species (RONS) to oxidize the lipids on the cell membrane to form pores and enter the cell. (3) Generally, the expression of aquaporin (AQP) in tumor cells increases, which is helpful for the transfer of RONS into cells. (4) With the increase of RONS derived from CAP, the antioxidant defense system of tumor cells is overwhelmed, which limits its protective effect on oxidative stress. (5) The increase of intracellular RONS affects intracellular calcium homeostasis. Through the interaction with inositol triphosphate receptor (IP3-RR) and ryanoid receptor (RR), calcium ions flow into the cytoplasm from endoplasmic reticulum (ER), meanwhile, mitochondrial permeability transition pores (mPTP) are opened to make calcium ions flow into mitochondria through mitochondria-associated ER membranes (MAM), resulting in mitochondrial-dependent apoptosis. (6) The increase of intracellular RONS can activate many different mitogen-activated protein kinase (MAPK) cascades, resulting in cell apoptosis. (7) CAP induces DNA and RNA damage in tumor cells, including DNA single-strand or double-strand break (DSB, SSB), DNA-protein crosslinks (DPC), and chemical modification of DNA and RNA bases, resulting in apoptosis, necrosis and senescence. However, DSB may be the result of apoptosis induced by CAP rather than the direct effect of CAP. (8) The mechanism of other active components of CAP such as charged particles, electric field and ultraviolet radiation on tumor cells needs to be further studied. Created by Biorender.

On the one hand, studies have shown that ROS produced by CAP can oxidize lipids in the cell membrane and tend to induce formation of pores with a size of approximately 15 Å. These pores allow different free active substances to permeate the cell. For normal cells, the high cholesterol content in the plasma membrane prevents the entry of hydrogen peroxide and other active substances (46). However, the cholesterol level in the cancer cell membrane is lower than that of healthy cells; therefore, cancer cells are more vulnerable than normal cells to the influence of cell membrane lipid oxidation, which ultimately produces RONS that penetrate the cell, promote oxidative stress and induce apoptosis (47, 48) (Figure 2, 2).

**Figure 3:**
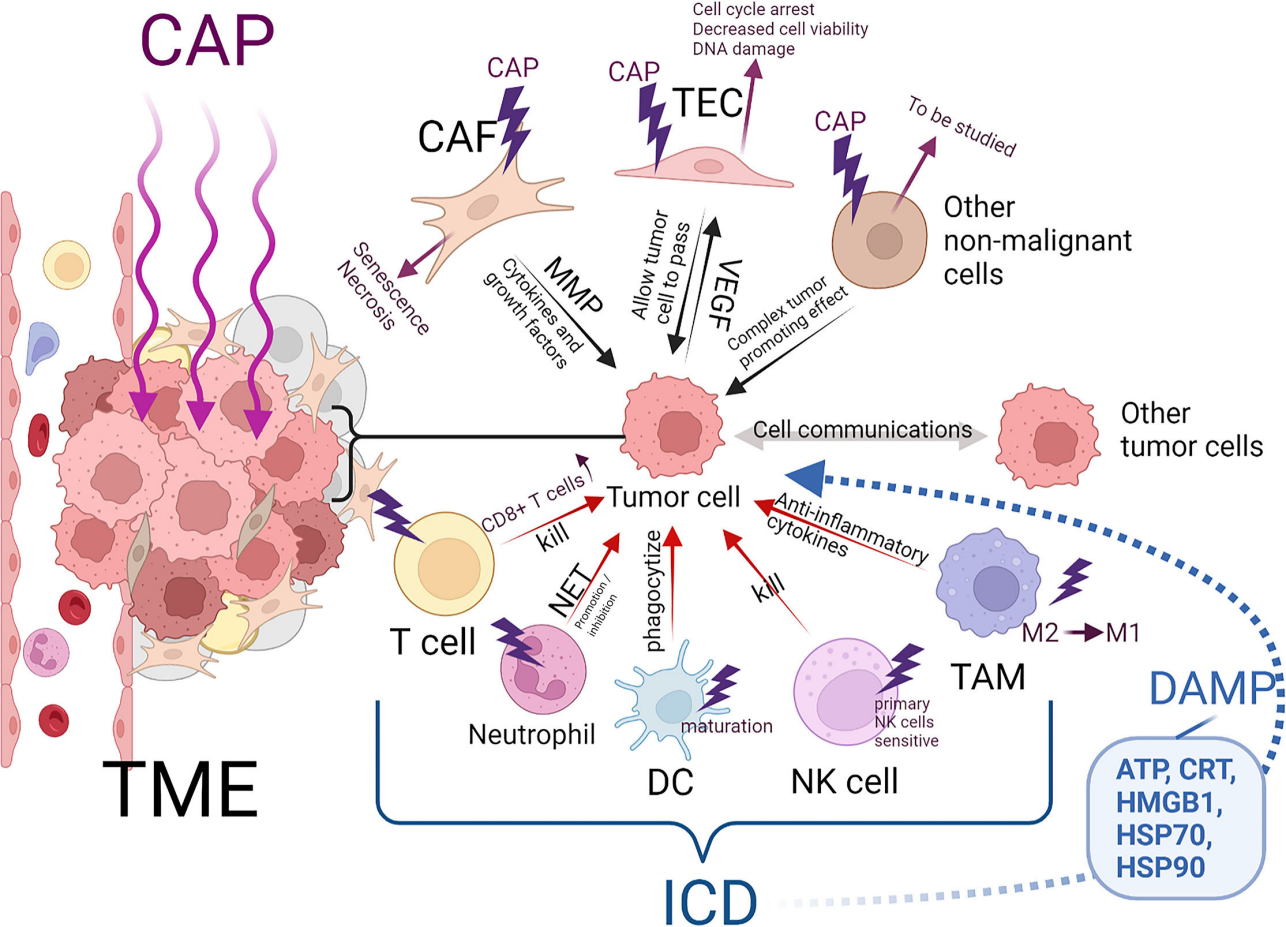
Effects of CAP on non-malignant cells in solid tumor microenvironment and induce immune death of tumor cells. Created by Biorender.

On the other hand, cancer cells tend to express more AQPs in cytoplasmic membranes, enabling cancer cells to absorb H_2_O_2_ produced by plasma exposure faster than normal cells (45) (Figure 2, 3). AQP is a tetramer that forms a key channel in the cell membrane (49) that promotes the transmembrane transport of H_2_O_2_ and other small molecules, including carbon dioxide, NO, ammonia, urea and glycerol. (50, 51) Currently, three AQPs (AQP1, AQP3 and AQP8) are known to be involved in the transport of H_2_O_2_ in cells (52) Yusupov et al. performed computer simulation to determine the selective mechanism of AQP action in tumor cells, and the mechanism was found to be triggered by the high expression of AQPs in the cell membrane. (53).

The selection mechanism of the CAP-induced scavenging effect on RONS in the antioxidant defense system of tumor cells has not been thoroughly studied. However, studies showed that the interactions of singlet oxygen produced by CAP triggered tumor cell production of higher concentrations of secondary singlet oxygen, resulting in the profound inactivation of protective catalase and SOD in tumor cells. (31, 43) In addition (15), the mRNA expression levels of GSTA4, MSRB3, SOD1, SOD2, CAT2 and HMOX1 in cholangiocarcinoma cells and human primary hepatocytes were compared in this study. Compared with those in the two cholangiocarcinoma cell lines, the mRNA expression of several RONS scavenging enzymes in the hepatocytes was significantly increased. In addition, other studies showed that the activity of ROS scavenging enzymes, including glutathione peroxidase, catalase and superoxide dismutase, decreased significantly after CAP treatment (54). However, CAP treatment increased the amount of nonenzymes, such as glutathione, which was significantly oxidized, in the antioxidant-reduction system of lymphocytes (55), while CAP treatment decreased the ratio of glutathione to glutathione disulfide (GSH/GSSG) and NADPH to NADP^+^ in cancer cells (54, 56). These observations suggest a potential defense mechanism that protects normal cells from exhibiting excessive ROS levels specifically by producing ROS scavenging enzymes and nonenzymes (Figure 2, 4).

### Pathways Triggered by CAP Active Compounds

Thus far, we have mainly summarized the oxidative damage mechanism of RONS derived from CAP on cells and discussed selective CAP effects on tumor cells. The active components of CAP are not only derived from RONS; charged particles, ultraviolet radiation and electromagnetic fields also exert effects (57). However, except for the mechanism of oxidative damage induced by RONS in cells, these mechanisms have not been elucidated. Therefore, herein we explore the most direct effects of RONS derived from CAP on cells, the known signaling cascades triggered by RONS, and their effects on cells.

As mentioned earlier, RONS derived from CAP causes lipid peroxidation of the tumor cell membrane, which leads to an increase in active substances, triggering signaling cascades inside and outside the cell. Calcium ions (Ca^2+^) are important second messengers involved in intracellular and extracellular signaling cascades and play key roles in cell apoptosis and senescence. There is a close association between calcium signaling and ROS signaling (58). One study showed that intracellular Ca^2+^ levels increased after direct and indirect CAP treatment of melanoma cells (59). They observed that CAP-induced Ca^2+^ influx was mainly derived from intracellular Ca^2+^ stores in endoplasmic reticulum (ER) and mitochondria. The specific mechanism was found to be related to the ryanodine receptor in the ER and mitochondrial permeability transition pores (mPTPs), and it was similar to the mechanism leading to increased intracellular Ca^2+^ levels induced by RONS. Notably, RONS regulate the cellular Ca^2+^ channel by modifying the sulfhydryl group of a cysteine residue. Ca^2+^ is released from the ER through ryanodine receptor and inositol 1,4,5-trisphosphate receptor mediation (60, 61), leading to ER stress and mitochondrial Ca^2+^ overload, oxidative stress, membrane depolarization and mPTP opening (62) by releasing cytochromec (63, 64). RONS induce intramolecular disulfide bond formation mediated by adenine nucleotide translocase, enhancing the sensitivity of mPTP to Ca^2+^ (65). Therefore, increased cytoplasmic Ca^2+^ realized through CAP is likely caused by RONS (59). Some studies have reported mitochondrial oxidation and membrane potential depolarization after CAP treatment, resulting in mitochondria-mediated apoptosis (55, 66), confirming the accuracy of the aforementioned mechanism (Figure 2, 5)

Moreover, it is clear that ROS can activate many different mitogen-activated protein kinase (MAPK) cascades, including stress kinase, c-Jun N-terminal kinase (JNK) and stress-activated protein kinase (SAPK) pathways (67). Among these kinases, extracellular regulated protein kinases (ERK) 1/2, JNK and p38 kinase have been the most extensively studied (68). Research has shown that plasma-derived RONS can induce the death of tumor cells via apoptosis and other forms of death by activating the ERK1/2, JNK and p38 MAPK signaling pathways (69, 70) (Figure 2, 6).

In addition, the increase in intracellular ROS can induce DNA damage (Figure 2, 7). The DNA damage caused by ROS includes single-strand breaks (SSBs) or double-strand breaks (DSBs) in DNA, oxidative damage, and DNA–protein crosslinking (DPC) (71–73). Some studies have evaluated cancer cell DNA strand breaks caused by CAP treatment by detecting the phosphorylated form of histone H2AX: γ H2AX (70, 74–76). The H2AX histone is a variant of histone H2A in mammalian cells, which has a specific serine glutamine motif in the carboxy terminus (C-terminus). PI3 kinase phosphorylates H2AX to generate γH2AX, which is the key response to DNA fragmentation (77). Therefore, γH2AX is considered by many scholars to be an important marker of DNA damage and repair. DSB activates ATM [a DNA damage response kinase in the PI3K-like protein kinase family (78)], which phosphorylates histone H2AX and activates P53-mediated signaling pathways and TP73 (a tumor suppressor protein), leading to apoptosis. Increases in ATM, P53 and TP73 have also been observed in several tumor cells (oral squamous cell carcinoma, lung adenocarcinoma, melanoma, etc.) treated directly or indirectly by CAP (74, 79, 80). In addition, studies have proven that CAP causes DNA strand breaks in cancer cells in a time-dependent manner, and the specific time is related to different CAP-generating devices (75, 81–83). Despite the support offered by the aforementioned studies, some recent studies have suggested that DNA strand breaks may not be direct effects of ROS produced by CAP but results of the cell death induced by CAP (84, 85). For example, Guo et al. studied the effect of CAP on DPC in bacteria, yeast and human cervical cancer cells (71). Plasma-derived RONS, including OH, ^1^O_2_, O^2−^ and ONOO-, reacted with proteins to form protein-free radicals, such as protein-C·, protein-O· and protein-OO·. Moreover, these protein-free radicals were unstable and reacted quickly with other functional groups to form alcohols, carbonyl groups and hydroperoxides. These products were converted into protein-free radicals before they reacted with DNA and RNA to form covalent cross-links, resulting in cell death. Moreover, direct irradiation through CAP led to the chemical modification of DNA bases, specifically, the production of 8-oxoguanine (8-oxoG) (86). The 8-oxoG DNA damage is more easily induced than strand breaks. However, due to the upregulation of intracellular DNA repair enzymes, this DNA damage may not be the main cause of cell death (87). Nevertheless, Cheng et al. demonstrated that 8-oxoG oxidative modification of histone mRNA followed by S-phase degradation and chromatin instability in the early stage of the cell cycle was a key mechanism of CAP-induced cell death in breast cancer cell lines (85).

The mechanisms of ROS-induced cell death have been extensively studied by many teams, but little research on the corresponding RNS mechanisms have been reported. As mentioned in the previous section, excessive NO derived from RNS may inhibit tumor formation. NO derived from RNS can interact with superoxide anions derived from NOX, resulting in the formation of nitrous peroxide. After protonation of peroxynitrite through the action of proton pumps located in the cell membrane, the resultant peroxynitrous acid may decompose, resulting in the formation of nitrogen dioxide and apoptosis-inducing hydroxyl radicals. This may cause inactivation of tumor cells. One study showed that long-term exposure of cells to NO and/or peroxynitrite may deplete the intracellular glutathione pool and make mitochondria more vulnerable to the harmful effects of ROS (88). The interaction between RNS and components of the electron transport chain can increase the production of reactive oxygen species in mitochondria, thus triggering the mechanism leading to cell death.

However, a recent study showed that known RONS, such as singlet oxygen, hydrogen peroxide, NO and nitrite/nitrate, are not the main causes of plasma-activated medium (PAM)-mediated selective cell death in the Hep3B and Huh7hepatocellular carcinoma cell lines. The results showed that other factors (such as negatively charged particles) were involved in the physiological effects of CAP and PAM (89). Currently, the effects of other active components produced by CAP (such as free charged particles and electric fields) on tumor cells have not been studied in-depth (Figure 2, 8). How these components change the intracellular environment and how the cellular response and regulatory mechanisms respond to these changes remain unclear. (90) Nevertheless, many studies have reported that nanosecond-pulsed electric fields can lead to contraction and death of tumor cells in cancer tissue (91–93). In summary, the active components of CAP and their specific mechanisms need to be further studied.

## Mechanisms Of Cap Effects On The Tme

Understanding the effect of CAP on solid tumors, the relationship between CAP and the TME clearly cannot be ignored. The TME is composed of malignant and nonmalignant cells, tumor blood vessels and the ECM, all of which constantly interact with each other. Nonmalignant cells in the TME are dynamically involved in all stages of carcinogenesis, and they usually promote tumors at these disease stages. These nonmalignant cells include immune cells, endothelial cells, fibroblasts, and adipocytes (94). In addition to cells, the TME also includes ECM components, the most prominent of which are collagen, fibronectin, polysaccharide chain, glycoprotein and proteoglycan (14). Cells and extracellular matrix communicate closely through the dynamic network of soluble factors such as cytokines, chemokines, growth factors, angiogenic factors and enzymes to coordinate uncontrolled cell growth, resistance to cell death, hypoxia tolerance and tumor dysplasia. These interactions are necessary for the formation of new blood vessels and lymphatic vessels, stroma remodeling, recruitment of immune cells and cancer-related fibroblasts, and metastasis (14, 17).

### Effects of CAP on Nonmalignant Cells

In cancer, activated fibroblasts (called cancer-associated fibroblasts, CAFs) are thought to play key roles in malignant progression. CAFs promote tumor development by secreting matrix metalloproteinases (MMPs) to initiate ECM remodeling and secrete a variety of cytokines and growth factors (95). A study showed that plasma exerted dual effects on fibroblasts; that is, short-term treatment promoted cell activity and collagen production, and long-term treatment inhibited cell activity and collagen production (96). Higher concentrations of CAP-derived ROS and longer plasma treatment can induce the senescence and necrosis of fibroblasts (97, 98).

The two main types of immune cells in the tumor immune microenvironment (TIME) are tumor-associated macrophages and T lymphocytes. Other cells, including B lymphocytes, natural killer cells (NK cells) and dendritic cells (DCs), are also involved in the immune escape of tumor cells. Recently, many studies have proven the effect of CAP on tumor-associated immune cells, and these studies are described in the following section.

In the process of tumor formation, a tumor-specific vascular system emerges, and the newly formed blood vessels provide oxygen and nutrients to the tumor, thereby supporting tumor progression and providing a channel for tumor cell metastasis (99). Among that of the tumor cells involved in angiogenesis, the role of tumor endothelial cells (TECs) is very important. Vascular endothelial growth factors (VEGFs) secreted by tumor cells loosen tight junctions between endothelial cells (100), resulting in vascular infiltration, and excess TECs, to which other cells associated with normal vascular structures cannot readily adhere (99, 101). The intercellular gaps formed by these mechanisms allow macromolecules and tumor cells to pass freely into growing vessels (94). One study showed that endothelial cells exhibit higher levels of double-strand DNA damage than keratinocytes or fibroblasts after CAP irradiation (102). Moreover, higher levels of ROS derived from CAP can induce cell cycle arrest and decrease cell viability and thus reduce DNA damage in vascular endothelial cells (103). These findings support the supposition that CAP clearance of tumor endothelial cells may help control tumor progression.

Other cells such as adipose-derived stem cells (AMSCs) promote angiogenesis, antiapoptosis, proliferation and pluripotent differentiation, which are usually associated with tumor initiation and metastasis (104). However, to date, research on the effect of plasma on AMSCs in a tumor background is very limited.

### Effects of CAP on a Cellular TME Components

In addition to the aforementioned cells, the TME is composed of collagen, elastin, fibronectin, glycoproteins and proteoglycans, collectively referred to as the ECM (14). Due to the overgrowth of tumor cells in the TME, the ECM in tumors usually shows excessive collagen deposition (105). CAP can inhibit tumor progression by oxidizing or destroying the structure of ECM (Figure 2, 1). Two studies on the clinical application of CAP in the treatment of head and neck cancer revealed that connective tissue proliferation induced by CAP after wound treatment indicated an increase in collagen deposition (106, 107). This outcome may be related to the aforementioned effect of CAP on fibroblasts. Another study showed that CAP inhibited the excessive synthesis of collagen in keloid fibroblasts (KFs) (108), and the activated fibroblasts may have similar characteristics with CAFs (109). An *in vitro* study on the effect of CAP on dentin surfaces and extracted type I collagen fibrils showed that CAP can destroy the structure of collagen (110). However, it remains to be seen whether CAP has any effect on the structure of tumor-related collagen fibers. Integrin is an adhesion molecule on the cell surface that plays a vital role in the adhesion, migration and invasion of tumor cells (111). The inhibitory effect of CAP on the adhesion, migration and invasion of tumor cells has been observed. In addition, after CAP treatment, the expression of integrin α2, integrin α4 and focal adhesion kinase (FAK) on the surface of melanoma cells has been found to be inhibited (112). Similarly, another study showed that CAP treatment inhibited the expression of integrin β1 and integrin αv in mouse skin cancer cells (113). These outcomes indicate that CAP may inhibit the adhesion, migration and invasion of tumor cells by inhibiting the expression of integrins. In addition, ROS can destroy or oxidize other ECM factors, such as hyaluronic acid (HA, an ECM proteoglycan) and fibronectin (114, 115). However, the effect of CAP on these proteins has not been extensively studied.

### Effects of CAP on Cell Communication

Plasma affects the communication between cells and between cells and the ECM. Some untreated cells outside the circumferential area of diffused ROS derived from CAP-treated cells can be damaged by plasma treatment. This outcome can be explained by the bystander effect and the abscopal effect (14). The bystander effect causes CAP-treated cells to transmit signals to untreated neighboring cells to induce biological changes. The effect of CAP on signaling pathways has been reported, and these damage signals can be transmitted through communication junctions [ion channels (59, 116), extracellular vesicles (117–119), gap junctions (120), and connexins (120–122)], occluding junctions [tight junctions (123), claudins (124), and occludins (124)] and anchoring junctions [adherens (122, 124–126) and desmosomes (127)]. As the current research on the effect of CAP on the malignant cell communication is not sufficient, the cellular communication references mentioned above include the study of malignant cells and non-malignant cells. In their review (14), Privat et al. described in detail the bystander effect mechanism in CAP-treated cells. Moreover, the abscopal effect enables CAP-treated cells to initiate responses in cells far from the treated area; that is, it is a systemic response involving the immune system (128). Plasma has been shown to inhibit the growth of melanoma tumors in distant untreated areas in mice, indicating that plasma therapy affects the immune response (129). The immune-related response induced by CAP therapy applied to tumors is described in the next section.

## Cap Triggers Icd And Affects Tumor-Related Immune Cells

On the basis of recent research, a new mechanism of CAP-induced tumor cell death has been proposed: CAP causes ICD (130–134). Interestingly, in addition to ICD, plasma can kill tumor cells by directly acting on tumor-related immune cells (Figure 3).

First, we discussed the effect of CAP on tumor-related immune cells. The key participants in the antitumor immune response are antigen-presenting cells (APCs), T cells and natural killer cells (NK cells) (135). Of course, other cells, such as neutrophils, are involved (136). APCs mainly include two kinds of cells, namely, macrophages and DCs. During tumor progression, tumor cells induce tumor-associated macrophages (TAMs) to differentiate into protumor type macrophages (with M2-like polarization), not into proinflammatory type macrophages (with M1-like polarization), which facilitate kill cancer cells. (137, 138) CAP can induce the M1 polarization of THP-1 macrophages derived from monocytes. These macrophages secrete many anti-inflammatory cytokines (IL-1α, IL-1β, IL-6, TNF-α, etc.) that kill tumor cells (130, 139). Macrophages activated by CAP showed greater mobility and tumor infiltration ability (130, 133). In addition, M1 polarized macrophages can reduce the migration and invasion ability of cancer cells (139). In the later stage of carcinogenesis, the epithelial-mesenchymal transition (EMT) contributes to many malignant characteristics of cancer cells, including antiapoptotic, migration, invasion and stem cell-like characteristics (140). M1 polarized macrophages may delay the EMT process and increase the expression of E-cadherin in glioma cells *in vitro* and *in vivo*, thus affecting the migration and invasion of glioma cells (139). DCs, which are APCs, play important roles in antigen processing and presentation to T cells, which induces an immune response to the tumor cells. However, because tumor cells release factors that inhibit or reverse the maturation and normal function of DCs, DCs can be transformed into tumor-associated DCs (TIDCs), and adaptive immunity is blocked through a variety of mechanisms (141, 142). In a study of plasma therapy on pancreatic tumors in mice, plasma therapy did not affect the number of TIDCs. (143) However, another *in vitro* study showed that DCs were more likely to phagocytize pancreatic tumor cells treated indirectly by plasma because these cells expressed and released damage-associated molecular patterns (DAMPs) that characterize ICD and promote DC maturation (144). Studies on long-term survivors of pancreatic cancer revealed that the patient's T cell bank is key to the fight against cancer (145). A significant increase in T cell and T cell infiltration has been observed in mouse pancreatic tumors exposed to plasma-activated medium (143). Furthermore, CAP-derived RONS can upregulate the expression of major histocompatibility complex-1, benefiting antigen presentation by cancer cells that may lead to an increase in CD8+ T cells in tumors, increasing the rate of tumor cell death (146, 147). Considerable evidence has shown that an increase in neutrophils in peripheral blood and tumors of cancer patients is related to poor clinical results (136). However, only one study on wound healing reported that plasma induced the formation of neutrophil extracellular traps (NETs) and the secretion of IL-8, which may have contributed tumor treatment effects (148). Primary NK cells are as sensitive as adaptive lymphocytes to plasma therapy, but activated NK cells are not (149).

Additionally, plasma therapy can cause ICD in tumors. ICD is related to the ability of the immune system to induce an effect similar to that of DC-based anticancer vaccines (155). ICD produces several DAMPs, including adenosine triphosphate (ATP), calreticulin (CRT), high mobility group protein B1 (HMGB1), heat shock protein (HSP) 70, and HSP90 (156–159). These molecules are directly exposed on the cell surface or secreted by cells undergoing autophagy, ER stress or ROS-induced oxidative stress (160–163). When plasma irradiates tumor cells, DAMP signals are emitted (132). Extracellular ATP acts as a “find me” DAMP signal, recruiting nearby immune cells and activating them (164). CRT exposed on the surface of the cell plasma membrane (ecto-CRT) is an “eat me” signal and mediates the phagocytosis of tumor cells by APCs (159). ICD has been observed in studies of many tumor cell types, including pancreatic cancer, colorectal cancer, lung cancer and malignant melanoma cells, treated directly or indirectly *in vitro* by plasma (10, 107, 131, 132, 144, 147, 150–154). Lin et al. proposed that the main effective components of plasma-induced ICD are charged and/or short-lived ROS, including 1O2, ∙OH and O2∙- (10, 132). Nevertheless, other active CAP components and the specific mechanism by which they induce ICD need to be studied further (Table 1).

**Table 1:** The studies of CAP in the treatment of tumors mentioned in the review.

**Tumor types**	**Direct/indirect**	**Type of study**	**Plasma source**	**References**
The HNSCC cell lines (JHU-022, JHU-028, JHU-029, SCC25)	Direct	*In vitro*	Helium-based plasma jet (created by The George Washington University)	(8)
OSC 19 cell lines and FaDu cell lines	Direct	*In vitro*	MiniFlatPlaSter^®^ (the Max Planck Institute for Extraterrestrial Physics)	(9)
B16F10 murine melanoma cell lines and A375 human melanoma cell lines	Direct/indirect	*In vitro*	Microsecond-Pulsed DBD Plasma (advanced plasma solutions)	(10)
HuCCT1 cell lines and EGI-1 cell lines	Direct/indirect	*In vitro/in vivo* (mice model)	Helium-based plasma jet (laboratory self-made)	(15)
MDA-MB-231, SW480, MCF-7, PC3 and NCF3 cancer cell lines	Direct	*In vitro*	Argon-based plasma jet kINPen 11 (neoplas, Germany)	(16)
Head and neck squamous cancer	Direct	*In vivo* (clinical)	Argon-based plasma jet (kINPen MED, neoplas tools GmbH, Germany)	(24)
Murine melanoma cell lines (B-16)	Direct	*In vitro/in vivo* (mice model)	Elongated flexible CAPJ (laboratory self-made)	(28)
Human gastric adenocarcinoma cell lines (MKN-45, ACC 409)	Indirect	*In vitro*	‘Corona pen' plasma source (laboratory self-made)	(31)
Human hepatoma cancer cell lines (HepG2)	Direct	*In vitro*	Atmospheric pressure room temperature plasma jet (laboratory self-made)	(54)
Human cancer cell lines glioblastoma (T98G), thyroid carcinoma (SNU80) and oral carcinoma (KB)	Direct	*In vitro*	Atmospheric pressure non-thermal DBD plasma (laboratory self-made)	(56)
Murine melanoma tumor cell lines (B16F0)	Direct	*In vitro*	Single-cellular-level sized microplasma jet (laboratory self-made)	(57)
Malignant melanoma cell lines (Mel Juso, Mel Im)	Direct/indirect	*In vitro*	miniFlatPlaSter (the Max Planck Institute for Extraterrestrial Physics)	(59)
Human cervical cancer HeLa cell lines (ATCC CCL-2) and lung cancer A549 cell lines	Direct	*In vitro*	Fabricated microplasma jet system (laboratory self-made)	(69)
Glioblastoma (T98G) and lung adenocarcinoma (A549) cell lines	Direct	*In vitro*	Soft plasma-jet system (laboratory self-made)	(70)
Human oral cavity cancer cell lines (MSK QLL1, SCC1483, SCC15, and SCC25)	Direct	*In vitro*	Spray-type non-thermal atmospheric plasma system (laboratory self-made)	(74)
Colorectal cancer cells (HCT116)spheroids	Indirect	*In vitro*	Helium-based plasma jet modified from DBD (laboratory self-made)	(75)
Murine (B16) and human (SK-MEL-28) melanoma cell lines	Direct	*In vitro*	Argon-based plasma jet kINPen	(76)
Human lung adenocarcinoma (A549) cell lines	Indirect	*In vitro*	Argon-based plasma jet (laboratory self-made)	(79)
Human melanoma cell lines (Mel007)	Direct	*In vitro*	Helium-based plasma jet (laboratory self-made)	(80)
Oral cancer cell lines (SCC-25)	Direct	*In vitro*	Nitrogen-based plasma jet (laboratory self-made)	(81)
Glioma cell lines (LN18, LN229 and U87MG)	Direct	*In vitro*	Surface Micro Discharge (SMD) plasma device	(82)
Breast cancer cell lines (BT-474, SK-BR-3, MCF-7 and MDA-MB-231)	Direct	*In vitro*	Canady Helios Cold Plasma™ (CHCP)	(85)
Human hepatoma cell lines (Hep3B and Huh7)	Indirect	*In vitro*	s-DBD device (laboratory self-made)	(89)
Locally advanced cancer of the oropharynx (pT4)	Direct	*In vivo (clinical)*	Argon-based plasma jet (kINPen MED)	(106)
Human pancreatic adenocarcinoma cell lines (Colo-357, PaTu8988T) and murine pancreatic cancer cell lines (6606PDA)	Direct	*In vitro/in vivo (mice model)*	Argon-based plasma jet (kINPen 09)	(118)
Human ovarian cancer cell lines (OVCAR-3 and SKOV-3)	Direct	*In vitro*	Argon-based plasma jet kINPen	(119)
Murine melanoma tumor cell lines (B16F10)	Direct	*In vivo* (mice model)	Nanosecond pulsed streamer discharge (laboratory self-made)	(129)
Human glioblastoma multiforme (T98G) and lung adenocarcinoma (A549) cell lines	Direct	*In vitro*	μ-DBD plasma device (laboratory self-made)	(130)
Human nasopharyngeal carcinoma (CNE-1) cell lines	Direct	*In vitro*	Nanosecond pulsed DBD plasma (laboratory self-made)	(131)
Human lung adenocarcinoma (A549) cell lines	Direct	*In vitro*	Nanosecond pulsed DBD plasma (laboratory self-made)	(132)
Human astrocytoma (U251MG and U87MG) cells spheroids	Direct	*In vitro/in vivo* (mice model)	μ-DBD plasma device (laboratory self-made)	(139)
Murine pancreatic cancer cell lines (6606PDA)	Indirect	*In vivo* (mice model)	Argon-based plasma jet (kINPen MED)	(143)
Human pancreatic cancer cell lines (MIA-Paca-2, PANC-1, BxPC3, and Capan-2)	Indirect	*In vitro*	Argon-based plasma jet (kINPen MED)	(144)
Murine melanoma tumor cell lines (B16F10)	Direct	*In vitro*	Argon-based plasma jet (kINPen 11)	(147, 150)
Murine colon carcinoma (CT26, MC38) and pancreatic cancer (6606PDA) cells spheroids	Indirect	*In vitro/in vivo* (mice model)	Argon-based plasma jet (kINPen)	(151)
Murine colon carcinoma (CT26) cell lines	Direct	*In vitro/in vivo* (mice model)	Nanosecond pulsed DBD plasma (laboratory self-made)	(152)
Human PDAC (PANC-1) and melanoma (Hmel1 MM, HBL MM) cell lines	Indirect	*In vitro*	PetriPlas source (designed at INP)	(153)
Murine colon carcinoma (CT26) cell lines	Direct	*In vitro*	Argon-based plasma jet (kINPen)	(154)

## Conclusions

We reviewed the currently known mechanisms of CAP effects on solid tumors. First, we introduced the currently used plasma treatment methods in tumors. Then, we analyzed the active components of CAP, including free charged particles, ROS and reactive nitrogen free radicals, ultraviolet radiation and electric fields, and reviewed the selectivity of RONS, the main active components of CAP, with respect to tumor cells and the mechanisms associated with oxidative damage. The selectivity of the CAP effects on tumor cells is mainly reflected by the following observations: 1. ROS produced by plasma can oxidize the plasma membrane, and the low cholesterol content in the tumor cell membrane increases tumor cell membrane permeability. 2. The high levels of AQPs in the tumor cell membrane makes absorption of ROS by tumor cells easier. 3. CAP treatment inactivates the antioxidant defense system in tumor cells. The following mechanisms of plasma-derived ROS-induced oxidative damage in cells have been elucidated to date: 1. Increased intracellular calcium signals and mitochondrial calcium overload cause ER stress, inducing mitochondrial-mediated apoptosis; 2. activation of different MAPK signaling cascades induces apoptosis; and 3. RNS and ROS may exert a synergistic effect in damaging DNA and RNA in cells. Next, we described the important role of CAP in nonmalignant cells and the ECM in the TME. In addition, the role of CAP in tumor immunity cannot be ignored. CAP destroys tumors by activating tumor immune cells and inducing ICD in tumors.

To sum up, CAP has many effects on solid tumors, and these effects are dose-dependent. The effects of optimal dose CAP on solid tumors are mainly inhibition. (The optimal dose cannot be determined because there is no standardized CAP equipment). These effects include killing tumor cells, inhibiting non-malignant cells and ECM in TME, affecting the communication between tumor cells, and inducing immunogenic death of tumor cells.

Currently, the mechanisms of CAP treatment effects on solid tumor are still largely unclear, and the recent research has been limited mainly studying the oxidative stress response induced by the RONS produced by CAP in tumor and related cells. However, one study showed that known RONS may not be the main components involved in the killing effect of CAP on tumors (89); therefore, studying other active components of CAP (such as charged particles) is critical. Currently, 3D models of TME components (such as spheroids, organoids, scaffolds, tumor-on-a-chip and 3D bioprinted tumors) can be used to simulate solid tumors *in vitro* and thereby determine the effect of CAP on solid tumors. In addition, the combination of plasma and nanoparticles (NPs) is a positive research direction (165). NPs have shown great potential for local binding with the RONS produced by CAP (166), and CAP enhances NP delivery and increases RONS levels in target tissue (167–169). Although many teams have explored these aspects of CAP treatment, the clinical application of this combination therapy needs to be further studied.

## Author Contributions

TM, XX, CD, and HZ were the major contributors in writing the manuscript. TM drafted the main part of the manuscript. XX contributed to literature retrieval and manuscript revision. KR contributed to the collection and collation of documents. TS revised the content of tumor microenvironment. HW revised the content of tumor immunity. CD and HZ proposed different amendments to the manuscript and standardizes the format of the paper. All authors read and approved the final manuscript.

## Funding

This review was supported by the Natural Science Basic Research Program of Shaanxi (Program No. 2019JQ-948). The funding body had no role in the design of the study and collection, analysis, and interpretation of data and in writing the manuscript.

## Conflict of Interest

The authors declare that the research was conducted in the absence of any commercial or financial relationships that could be construed as a potential conflict of interest.

## Publisher's Note

All claims expressed in this article are solely those of the authors and do not necessarily represent those of their affiliated organizations, or those of the publisher, the editors and the reviewers. Any product that may be evaluated in this article, or claim that may be made by its manufacturer, is not guaranteed or endorsed by the publisher.

## Abbreviations

CAP: cold atmospheric plasma
ROS: reactive oxygen species
RNS: reactive nitrogen species
RONS: reactive oxygen and nitrogen species
ECM: extracellular matrix
TME: tumor microenvironment
ICD: immunogenic cell death
DBD: dielectric barrier discharge
PAW: plasma-activated water
NOX: NADPH oxidase
ADP: aquaporin
SOD: superoxide dismutase
ER: endoplasmic reticulum
mPTP: mitochondrial permeability transition pore
MAPK: mitogen-activated protein kinase
JNK: c-Jun N-terminal kinase
SAPK: stress-activated protein kinase
ERK: extracellular regulated protein kinases
SSB: single-strand break
DSB: double-strand break
DPC: DNA–protein crosslinking
8-oxoG: 8-oxoguanine
PAM: plasma-activated medium
CAF: cancer-associated fibroblast
TIME: tumor immune microenvironment
DC: dendritic cell
TEC: tumor endothelial cells
ASMC: adipose-derived stem cell
APC: antigen-presenting cell
EMT: epithelial-mesenchymal transition
TIDC: tumor-associated dendritic cell
DAMP: damage-associated molecular pattern
CRT: calreticulin
NP: nanoparticle.
